# Maternal Serum Ferritin Levels in Third Trimester and Risk of Small for Gestational Age in Northern Thailand: Implications for Management in Pregnancy

**DOI:** 10.3390/nu17243911

**Published:** 2025-12-13

**Authors:** Pak Thaichana, Ampica Mangklabruks, Amaraporn Rerkasem, Antika Wongthanee, Suthathip Wongsrithep, Kittipan Rerkasem

**Affiliations:** 1 Office of Research Administration, Chiang Mai University, Chiang Mai 50200, Thailand; pak.th@cmu.ac.th; 2 Research Institute for Health Sciences, Chiang Mai University, Chiang Mai 50200, Thailand; amaraporn.rer@cmu.ac.th (A.R.); antika.wong@cmu.ac.th (A.W.); suthathip.w@cmu.ac.th (S.W.); 3 Department of Internal Medicine, Faculty of Medicine, Chiang Mai University, Chiang Mai 50200, Thailand; ampica.m@cmu.ac.th; 4 Clinical Surgical Research Center, Department of Surgery, Faculty of Medicine, Chiang Mai University, Chiang Mai 50200, Thailand

**Keywords:** ferritin, birth outcomes, small for gestational age, placental weight, iron status

## Abstract

**Background:** Iron deficiency anemia is a recognized pregnancy risk, but excessive iron may also have adverse effects. Few studies, particularly in Asian populations, have examined elevated maternal ferritin in relation to birth outcomes. **Objectives**: To investigate the dose–response relationships between maternal serum ferritin concentrations in late pregnancy and birth outcomes, including preterm birth, small for gestational age (SGA), and placental characteristics. **Methods**: A retrospective study of 362 mother–infant pairs were analyzed. Maternal serum ferritin levels measured at 30–34 weeks’ gestation were divided into quintiles, using 30.1–43.0 µg/L as the reference. Logistic and linear regression models were applied to examine associations with preterm birth, SGA, and placental indices. **Results**: Preterm birth rates ranged 7.3–18.8% across ferritin quintiles, but no significant association was observed. In contrast, SGA prevalence increased from 20.8% to 47.2% (*p*-trend = 0.001). Women in the highest ferritin quintile had 3.31-fold higher adjusted odds of SGA (95% CI: 1.51–7.28, *p* = 0.003). Each SD increase in ferritin corresponded to 31% higher odds of SGA (OR = 1.31, 95% CI: 1.01–1.71). Elevated ferritin (>43.0 µg/L) was also associated with reduced placental weight (<415 g) (adjusted OR = 3.02, 95% CI: 1.61–5.69, *p* = 0.001). **Conclusions**: Increasing maternal ferritin levels in the third trimester were associated with a dose-dependent rise in SGA risk and reduced placental weight. These findings suggest that excessive iron status may adversely influence placental function and fetal growth, underscoring the need for individualized nutrition management during pregnancy.

## 1. Introduction

The first one thousand days of life, spanning from conception to two years of age, represent a critical window that profoundly influences long-term health trajectories [1]. Within this vulnerable period, maternal nutrition during pregnancy plays a foundational role in shaping fetal development and establishing health outcomes that extend well into adulthood [2]. Iron nutrition exemplifies this concept, as maternal iron status during pregnancy not only affects immediate birth outcomes but also sets the stage for neurodevelopmental, metabolic, and cardiovascular health across the lifespan [3].

Iron deficiency remains one of the most prevalent nutritional challenges during pregnancy, affecting approximately 40% of pregnant women worldwide [4]. The substantial increase in iron requirements during gestation—necessary to support placental development, fetal growth, and maternal red blood cell expansion—places pregnant women at heightened risk of inadequate iron stores [5]. Serum ferritin, the most reliable biomarker of iron storage, has emerged as a critical indicator for assessing maternal iron status, yet its interpretation presents unique clinical challenges during pregnancy [6].

Traditionally, clinical focus has centered on preventing iron deficiency and its associated complications. However, emerging evidence reveals a more complex relationship, suggesting that both abnormally low and elevated ferritin levels during pregnancy are associated with adverse outcomes [7]. Low maternal ferritin indicates iron deficiency and has been consistently linked to increased risks of preterm birth and low birthweight [8,9].More intriguingly, elevated ferritin levels have also been associated with adverse outcomes, including small for gestational age (SGA) births and preterm delivery, suggesting a U-shaped relationship between maternal iron status and fetal wellbeing [10,11].

SGA, defined as birth weight below the 10th percentile for gestational age, remains a critical global public health concern affecting over 20 million newborns annually [12,13]. Children born preterm or SGA face elevated risks of infant mortality as well as long–term cardiovascular disease, and metabolic disorders underscoring the far-reaching impact of intrauterine nutrition on health across the lifespan [14,15,16]

The relationship between maternal iron status and fetal growth appears to be mediated, at least in part, through placental function. The placenta serves as the critical interface between maternal and fetal circulation, adapting its structure and function in response to maternal nutritional status. Evidence suggests that maternal iron deficiency may trigger compensatory placental hypertrophy to maximize nutrient transfer to the developing fetus [17]. Conversely, elevated maternal iron levels have been associated with oxidative stress, potentially contributing to placental dysfunction and subsequently affecting fetal growth patterns [18]. Placental weight, as a key indicator of placental adequacy, may therefore serve as a crucial mediator linking maternal iron status to birth outcomes.

The timing of ferritin assessment during pregnancy is particularly important for understanding these relationships. While most prior studies have focused on early or mid-pregnancy [19,20], third-trimester assessment may offer unique insights for predicting birth outcomes and could potentially explain some of the inconsistent findings regarding optimal ferritin levels. The 30–34-week gestational age represents a critical period when fetal growth velocity is maximal and placental function is under peak demand, making it an opportune time for evaluating the impact of maternal iron status on pregnancy outcomes.

Given the complex and sometimes paradoxical relationship between maternal iron status and fetal growth, and the placenta’s central role as a mediator between maternal nutrition and fetal development, understanding the clinical implications of maternal ferritin assessment is essential. This study aimed to investigate the dose–response relationships between maternal serum ferritin concentrations at 30–34 weeks of gestation and birth outcomes including preterm birth, SGA, and placental characteristics to elucidate potential placental-mediated mechanisms underlying fetal growth restriction. The findings are expected to provide evidence-based insights into clinical practice and support the development of personalized nutrition strategies to optimize maternal and child health during the first 1000 days of life.

## 2. Materials and Methods

### 2.1. Study Design and Population

This retrospective study was conducted at the antenatal care (ANC) clinics and delivery units of two centers, Maharaj Nakorn Chiang Mai Hospital and Regional Health Promotion Center 1 Hospital Chiang Mai, the two largest providers of antenatal and delivery services in Northern Thailand at the time between 1989 and 1990 [21]. These two centers were the largest providers of antenatal and delivery services in the region, collectively managing more than 7000 deliveries annually during the study period. Of 2626 pregnant women were recruited at their first-trimester ANC visit (before 14 weeks). Initial informed consent was obtained, and sociodemographic/obstetric data were collected via questionnaires. Maternal anthropometrics and other data were recorded at each visit. A total of 442 women were excluded for the following reasons: delivered at other hospitals (*n* = 290), gestational age > 24 weeks upon re-examination (*n* = 9), twin pregnancies (*n* = 14), stillbirths (*n* = 11), abortions (*n* = 71), fetal deaths in utero (*n* = 15), and missing birth information (*n* = 32). Ultimately, 2184 mother–infant pairs with live births were included in the main cohort analysis, with 57.4% and 42.6% recruited from Maharaj Nakorn Chiang Mai Hospital and Regional Health Promotion Center 1 Hospital Chiang Mai, respectively. Simultaneously, a sub-study on maternal iron status and pregnancy outcomes was conducted within a selected subgroup of 462 women. Inclusion criteria were singleton pregnancies, routine antenatal care, and complete follow-up during the third trimester (median 32 weeks, range 30–34 weeks). Exclusion criteria were applied for incomplete iron profile measurements (*n* = 82), and unavailable placental characteristics (*n* = 18), 362 eligible mother–infant pairs were included in this analysis. Conducted by a consortium including RIHES, Chiang Mai University, and medical center staff, the study received approval from the Human Experimentation Committee (Research Institute for Health Sciences, Chiang Mai University). Due to its retrospective design, the ethics committees waived the requirement for written informed consent.

### 2.2. Maternal Iron Status Assessment and Outcome Definitions

Maternal data, including age, pre-pregnancy BMI, education, occupation, parity, smoking, and alcohol use, were collected via first-visit questionnaires. Gestational age was determined by last menstrual period and first-trimester ultrasound. Infant anthropometrics followed established methods [21]. Participants were followed until delivery. Fasting maternal blood samples were collected during the third trimester (median 32 weeks, range 30–34 weeks) to assess iron status indicators: ferritin, serum iron, total iron-binding capacity, transferrin saturation, mean cell hemoglobin concentration, and hemoglobin. Plasma was separated by centrifugation and stored at −20 °C. Iron profiles were analyzed at RIHES, Chiang Mai University. Maternal serum ferritin levels at 30–34 weeks of gestation were categorized into quintiles, using the fourth quintile (30.1–43.0 µg/L) as the reference group, to examine dose–response relationships. Moreover, maternal serum ferritin levels were categorized into two groups (≤43.0 and >43.0 µg/L) to specifically analyze the impact of elevated ferritin (>43.0 µg/L) on placental characteristics. Small for gestational age (SGA) was classified as a birth weight below the 10th percentile for gestational age according to INTERGROWTH-21st standards [22]. Preterm birth was defined as delivery before 37 completed weeks of gestation. Placental characteristics were categorized using standardized deviation scores calculated from gestational age-adjusted values. A consistent threshold was applied across all three parameters: levels within one standard deviation of the mean (−1 SD to +1 SD) were defined as Normal (for Placental Weight, Placental Diameter, and Umbilical Cord Length); levels falling more than one standard deviation below the mean (−1 SD) were classified as Low (for Placental Weight), Small (for Placental Diameter), or Short (for Umbilical Cord Length); and levels more than one standard deviation above the mean (+1 SD) were classified as High (for Placental Weight), Large (for Placental Diameter), or Long (for Umbilical Cord Length).

### 2.3. Laboratory Assessment

Iron status was evaluated using standardized laboratory methods following the recommendations of The International Committee for Standardization in Hematology [23]. Briefly, serum iron concentration was determined using the bathophenanthroline sulfonate method. All glassware was pre-treated to be iron-free. For the assay, 200 μL of serum was combined with 200 μL protein precipitant solution (trichloroacetic acid/thioglycolic acid in HCl). After 5 min at room temperature, samples were centrifuged (3500 rpm, 10 °C, 30 min). A 200 μL supernatant aliquot was then mixed with 200 μL chromogen solution (bathophenanthroline sulfonate). Following a 5 min color development, absorbance was measured spectrophotometrically at 535 nm against a reagent blank. Concentration was calculated from a ferrous ammonium sulfate standard curve (0.1–0.4 μg iron). The inter-assay coefficient of variation was <3.5%. For TIBC determination, 200 μL of plasma was saturated with 400 μL of chloride iron (0.5 mg iron/100 mL in 0.005 N HCl) and incubated for 5 min. Excess unbound iron was removed by adsorption with 50 mg of anhydrous magnesium carbonate powder. After vigorous shaking for 15 s initially and at three subsequent intervals over a 45–60 min period, samples were centrifuged at 3500 rpm at 10 °C for 35 min total (with gentle agitation after the first 5 min to dislodge precipitate from tube walls). A 200 μL aliquot of the clear supernatant was analyzed using the same procedure as for serum iron measurement. The intra-assay and inter-assay coefficients of variation were <4.0% and <5.0%, respectively. For hemoglobin determination, we conducted the following process: Pipet exactly 5.0 mL of cyanmethemoglobin reagent into each of 2 test tubes (No.1 and No.2). Add 0.02 mL of capillary, or well-mixed anticoagulated blood, to the cyanmethemoglobin reagent in test tube No.2. Rinse the pipet 3 to 5 times with the cyanmethemoglobin reagent until all blood is removed from the pipet. Mix the preceding solution well and allow it to stand at room temperature for at least 10 min to allow adequate time for the formation of the cyanmethemoglobin. Transfer the mixture to a cuvette and read in a colorimeter, or spectrophotometer, at a wavelength of 540 nm (or yellow–green filter) using the cyanmethemoglobin reagent in tube No.1 to blank the instrument (set percent transmittance at 100%). Record the reading for tube No.2 (patient’s unknown hemoglobin) from the percent transmittance scale and refer to the pre calibrated chart for the actual value of the hemoglobin in gram per dL.

### 2.4. Statistical Analysis

Continuous variables were presented as median with interquartile range (IQR: quartile 1, quartile 3), while categorical variables were expressed as frequencies and percentages. Group comparisons were performed using the Mann–Whitney U test for continuous variables and the chi-square test or Fisher’s exact test for categorical variables, as appropriate. To evaluate the association between maternal serum ferritin at 30–34 weeks’ gestation and adverse birth outcomes (preterm birth and small for gestational age [SGA]), ferritin levels were categorized into quintiles using the fourth quintile (30.1–43.0 µg/L) as the reference. Multiple logistic regression models were fitted to estimate crude and adjusted odds ratios (ORs) with 95% confidence intervals (CIs), including analyses for linear trend and per one-standard-deviation increase in ferritin. To address potential non-linear relationships, Non-linear relationships were examined using three approaches: a Wald test for the quadratic ferritin term, a likelihood ratio test comparing linear and quadratic models, and restricted cubic spline models with knots at the 5th, 35th, 65th, and 95th percentiles. Multivariable logistic regression models were adjusted for potential confounders including maternal age, pre-pregnancy BMI, maternal education, and parity. To further investigate associations with placental characteristics, ferritin was dichotomized at >43.0 µg/L, and multinomial logistic regression was used to model placental weight, diameter, and umbilical cord length categorized into low (<mean − 1 SD), normal (mean ± 1 SD, serving as reference), and high (>mean + 1 SD). Multinomial logistic regression models yielded odds ratios comparing low and high categories against the normal reference category. Models were adjusted for maternal age, pre-pregnancy BMI, maternal education, and parity, to account for their interrelationships. In addition, multiple linear regression models assessed relationships between placental parameters (continuous predictors) and standardized birth weight outcomes (INTERGROWTH-21st Z-scores and centiles). This model was adjusted for maternal age, pre-pregnancy BMI, maternal education, and parity. A backward stepwise selection method was employed to determine the most important factors associated with the outcomes. Variables with *p*-values greater than 0.2 were excluded from the model in each step, while those with *p*-values less than 0.05 were retained. *p*-values < 0.05 were considered statistically significant. Statistical analyses were performed using appropriate software. Statistical analyses were conducted using STATA version 17 (StataCorp, College Station, TX, USA), and statistical significance was defined as *p* < 0.05.

## 3. Results

### 3.1. Study Population Characteristics

**Figure 1:**
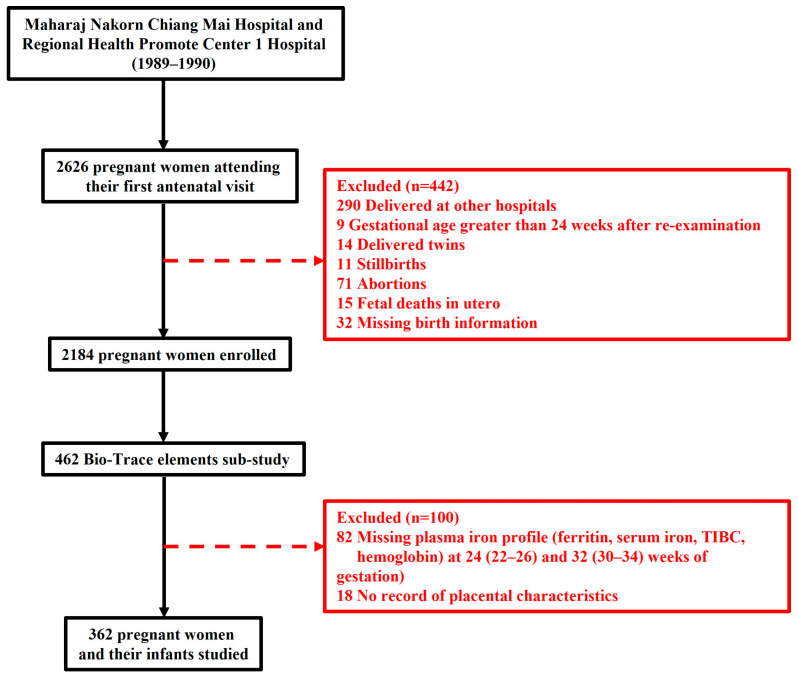
Flowchart of this study.

A total of 2626 pregnant women were initially enrolled in this study, which was carried out at Maharaj Nakorn Chiang Mai Hospital and Regional Health Promotion Center 1 Hospital, Chiang Mai, between 1989 and 1990. To investigate the pregnancy outcomes linked to trace elements throughout pregnancy, a total of 2184 pregnant women were included in the main cohort. Simultaneously, a sub-cohort study on maternal iron status and pregnancy outcomes was conducted using a convenience sampling selected subset of 462 women from the main cohort who had singleton pregnancies and attended routine antenatal care. Plasma samples were collected from these women during the third trimester (median 32 weeks, range 30–34 weeks of gestation) for iron profile measurements. Following the exclusion criteria, which accounted for delivered at other hospitals, multiple pregnancies, incomplete iron profile measurements, and other factors, 362 mother–infant pairs remained for final analysis (Figure 1).

As shown in Table 1, a total of 362 mother–infant pairs were included in the study cohort and compared with 1822 women from the non-study cohort. Among women in the study cohort, the majority (89.5%) were 20–35 years of age, with 7.5% younger than 20 years and 3.0% older than 35 years. Most participants (69.6%) had a normal pre-pregnancy BMI, while 12.2% were underweight, 10.2% overweight, and 8.0% obese. The largest occupational groups included unemployed women (30.1%), employees (17.7%), and agricultural workers (16.0%). Nearly two-thirds (65.2%) were nulliparous. Median gestational age at delivery was 39 weeks (IQR: 38–40), and median birthweight was 2850 g (IQR: 2420–3060). Male infants accounted for 52.2% of births, and cesarean section was performed in 10.5% of deliveries. When compared with the non-study cohort, maternal age, pre-pregnancy BMI, occupational were similar between groups (all *p* > 0.05). However, women in the study cohort were more likely to have completed only primary education (71.5% vs. 64.1%, *p* = 0.007) and to be nulliparous (65.2% vs. 56.3%, *p* = 0.002). Although gestational age at delivery did not differ between cohorts, infants in the study cohort had a significantly lower median birthweight (2850 g vs. 3050 g, *p* < 0.001). The proportions of male infants and mode of delivery were comparable between groups.

### 3.2. Maternal Iron Status Parameters and Their Association with Preterm Birth and Small for Gestational Age in Third Trimester

Our study investigated maternal iron status parameters, including ferritin, serum iron, total iron-binding capacity (TIBC), transferrin saturation, mean cell hemoglobin concentration (MCHC), and hemoglobin levels, at third trimesters. We examined the relationship between these parameters and adverse pregnancy outcomes, specifically preterm birth and small for gestation age (SGA). In Table 2, these parameters were stratified by birth outcomes: preterm versus term births and SGA versus non-SGA. A total of 362 pregnant women were included in this analysis. Among these participants, 45 women (12.4%) experienced preterm birth, while 317 women (87.6%) delivered at term. The median gestational age at delivery was 36 weeks (IQR: 35, 36) for the preterm birth group and 39 weeks (IQR: 38, 40) for the term birth group. Regarding fetal growth outcomes, 120 infants (33.2%) were classified as SGA, whereas 242 infants (66.8%) were appropriately grown (non-SGA). Maternal blood samples were obtained at approximately 32 weeks of gestation (range: 30–34 weeks) to evaluate iron status parameters. Serum ferritin levels revealed that women who subsequently delivered preterm had a median concentration of 31 µg/L (IQR: 17, 41; range: 5–149 µg/L), which was higher compared to those who delivered at term with a median of 23 µg/L (IQR: 14, 38; range: 3–312 µg/L). When stratified by fetal growth outcomes, mothers of SGA infants showed a median ferritin level of 26 µg/L (IQR: 17, 48; range: 3–312 µg/L), while mothers of non-SGA infants had a median of 23 µg/L (IQR: 12, 37; range: 3–270 µg/L). Serum iron concentrations demonstrated comparable patterns across groups. The preterm birth cohort exhibited a median serum iron of 101 µg/dL (IQR: 68, 127; range: 29–208 µg/dL), whereas the term birth group had a median of 94 µg/dL (IQR: 71, 112; range: 21–284 µg/dL). Similarly, mothers of SGA infants had a median serum iron of 96 µg/dL (IQR: 77, 117; range: 32–284 µg/dL) compared to 92 µg/dL (IQR: 68, 112; range: 21–236 µg/dL) in the non-SGA group. Total iron binding capacity (TIBC) values showed some variation between groups, with the preterm birth group demonstrating a median of 462 µmol/L (IQR: 420, 543; range: 277–822 µmol/L), which was lower than the term birth group at 497 µmol/L (IQR: 435, 561; range: 70–847 µmol/L). TIBC values for SGA and non-SGA groups were similar at 497 µmol/L for both, though with different interquartile ranges. Transferrin saturation percentages remained relatively consistent across all comparison groups, with median values of 18.6% (IQR: 14.2, 31.8; range: 5.1–66.7%) for preterm births and 18.3% (IQR: 13.8, 23.8; range: 5.0–100%) for term births. The SGA group showed a median transferrin saturation of 19.5% (IQR: 15.0, 24.6; range: 7.0–62.7%) compared to 17.7% (IQR: 13.3, 24.3; range: 5.0–100%) in the non-SGA group. Mean cell hemoglobin concentration (MCHC) and hemoglobin levels demonstrated minimal variation across all groups. MCHC values ranged from 32.0 to 32.2 across groups, while hemoglobin concentrations remained between 11.4 and 11.6 g/dL.

### 3.3. Association Between Serum Ferritin Levels and Birth Outcomes

Table 3 presents the evidence that elevated maternal serum ferritin levels in the third trimester are significantly associated with an increased risk of SGA in a dose-dependent manner. A total of 362 participants were included in the analysis, distributed across five quintiles based on serum ferritin levels at 30–34 weeks of gestation. The distribution was as follows: Quintile 1 (<13.0 µg/L, *n* = 77, 21.3%), Quintile 2 (13.0–20.0 µg/L, *n* = 72, 19.9%), Quintile 3 (20.1–30.0 µg/L, *n* = 77, 21.3%), Quintile 4 (30.1–43.0 µg/L, *n* = 64, 17.7%), and Quintile 5 (>43.0 µg/L, *n* = 72, 19.9%).

Preterm birth rates varied across ferritin quintiles (7.8–18.8%), with no consistent pattern observed. In the adjusted analysis using Quintile 4 as the reference, no significant associations were found across quintiles (*p*-trend = 0.484), and per-standard deviation increase in ferritin was not associated with preterm birth risk (adjusted OR = 1.01, 95% CI: 0.71–1.44).

In contrast, a significant dose–response relationship was observed between serum ferritin levels and SGA outcomes. For small for gestational age (SGA), the rates increased across ferritin quintiles, from 20.8% (Q1) to 47.2% (Q5), demonstrating a statistically significant trend (crude *p*-trend < 0.001; adjusted *p*-trend = 0.044). In the adjusted analysis using Quintile 4 as the reference, markedly elevated ferritin levels (Quintile 5: >43.0 µg/L) were significantly associated with increased SGA risk (adjusted OR = 3.31, 95% CI: 1.51–7.28, *p* = 0.003). Quintiles 1–3 showed numerically elevated odds ratios (Q1: OR = 1.06; Q2: OR = 1.72; Q3: OR = 2.04) but did not reach statistical significance (all *p* > 0.05), likely due to limited statistical power and overlapping confidence intervals. When modeled as a continuous variable, each standard deviation increase in serum ferritin was associated with 31% increased odds of SGA (adjusted OR = 1.31, 95% CI: 1.01–1.71, *p* = 0.044). Although the quintile-specific estimates suggested a possible U-shaped pattern, with elevated odds in both lower and higher ferritin categories, the wide and overlapping confidence intervals made visual interpretation inconclusive. Therefore, formal statistical testing was conducted to determine whether the ferritin–SGA association deviated from linearity. Formal testing for non-linearity using Wald test (*p* = 0.432), likelihood ratio test (*p* = 0.443), and restricted cubic spline analysis (*p* = 0.575) did not reveal significant departure from linearity (Supplementary Materials, Table S1).

### 3.4. Association Between Maternal Serum Ferritin Levels and Placental Characteristics

As shown in Table 4, we examined the association between elevated maternal serum ferritin (>43.0 µg/L vs. ≤43.0 µg/L) and placental characteristics. Placental parameters were categorized into three groups based on mean ± 1 SD, with the middle category serving as the reference group.

A significant threshold effect was observed for placental weight. Using multinomial logistic regression, women with elevated ferritin levels (>43.0 µg/L) showed significantly increased odds of having low placental weight (<415 g) compared to normal weight (415–629 g) (adjusted OR = 3.02, 95% CI: 1.61–5.69, *p* = 0.001). Conversely, no association was found between elevated ferritin and high placental weight (>629 g) (adjusted OR = 0.80, 95% CI: 0.33–1.93, *p* = 0.615). Neither placental diameter nor umbilical cord length demonstrated statistically significant associations with elevated maternal ferritin levels across any category (all *p* > 0.05).

### 3.5. Association Between Placental Characteristics and Infant Birth Weight

In Table 5, the result shows the associations between placental characteristics and infant birth weight outcomes. In the adjusted linear regression analysis, placental weight demonstrated a significant positive association with both birth weight Z-score (*β* = 0.39, 95% CI: 0.32 to 0.47, *p* < 0.001) and birth weight centile (*β* = 8.58, 95% CI: 6.78 to 10.37, *p* < 0.001). In contrast, placental diameter and umbilical cord length did not show statistically significant associations with either outcome. Specifically, placental diameter was weakly associated with birth weight Z-score (*β* = 0.02, 95% CI: –0.01 to 0.03, *p* = 0.075) and with birth weight centile (*β* = 0.24, 95% CI: –0.14 to 0.62, *p* = 0.219). Similarly, umbilical cord length showed no significant relationship with birth weight Z-score (*β* = 0.00, 95% CI: –0.01 to 0.01, *p* = 0.107) or centile (*β* = –0.15, 95% CI: –0.35 to 0.04, *p* = 0.121)

## 4. Discussion

This study elucidates the complex relationship between maternal iron status in late pregnancy and fetal growth outcomes. We identified a dose–response association between maternal serum ferritin concentrations at 30–34 weeks of gestation and the risk of small for gestational age (SGA) birth, with formal testing confirming no significant non-linearity. In contrast, no significant association was observed with preterm birth. In addition, a threshold effect was evident for placental weight, in which ferritin levels exceeding 43 µg/L were linked to an increased risk of low placental mass. Together, these findings suggest that elevated ferritin may impair fetal growth through placental-mediated mechanisms.

The most striking finding of our study is the dose–response relationship between maternal serum ferritin levels during 30–34 weeks of gestation and SGA risk. Women in the highest ferritin quintile (>43.0 µg/L) demonstrated 3.31-fold increased odds of delivering SGA infants compared to those in the reference quintile, with each standard deviation increase in ferritin associated with 31% increased SGA odds. Importantly, while the categorical analysis revealed varying odds ratios across quintiles (Q1: OR = 1.06, Q2: OR = 1.72, Q3: OR = 2.04, Q5: OR = 3.31), formal testing for non-linearity using three independent methods (Wald test: *p* = 0.432; likelihood ratio test: *p* = 0.443; restricted cubic spline analysis: *p* = 0.575) did not reveal significant departure from linearity (Supplementary Table S1). This indicates that the relationship can be adequately characterized as a progressive, monotonic increase in SGA risk with rising ferritin levels, rather than a U-shaped or J-shaped pattern. The apparent pattern observed in the categorical analysis reflects a dose–response relationship where only the highest ferritin levels (Quintile 5) reached statistical significance, likely due to the limited statistical power of categorical comparisons and the relatively modest effect sizes at lower ferritin ranges. These analyses comprehensively demonstrate that while categorical analysis shows varying point estimates, formal statistical testing supports monotonic linear characterization, validating both trend and per-SD analytical approaches.

Our findings align with recent studies across diverse populations worldwide [10,11,24], which consistently demonstrate associations between elevated ferritin–SGA, with effect sizes comparable to our observed odds ratio of 3.31. However, we advance this literature by confirming a monotonic dose–response relationship through formal non-linearity testing, strengthening evidence for continuous modeling approaches in meta-analyses. Our third-trimester assessment also extends prior early or mid-pregnancy studies [19,20], suggesting late-pregnancy ferritin better predicts birth outcomes.

Although data were collected from 1989–1990, the biological mechanisms linking iron status to placental function remain unchanged across eras. The key biological pathways implicated—altered placental iron metabolism and inflammation-related dysfunction [25], iron dysregulation leading to oxidative stress and impaired placental–fetal iron transfer [18,26], and disturbances in maternal–placental–fetal iron homeostasis supported by mechanistic and clinical biomarkers [5,27] operate through conserved physiological processes validated in contemporary populations. Recent studies consistently demonstrate similar dose–response patterns [10,11], confirming that these biological relationships transcend temporal context. Moreover, the pre-supplementation era in our cohort captures the full natural spectrum of maternal ferritin levels—from deficiency to physiological excess—without intervention-induced truncation observed in modern studies [28,29]. The lower obesity prevalence in our cohort (8.0% vs. 25–35% currently) [30,31], also minimizes confounding from chronic inflammation, allowing clearer assessment of ferritin’s independent effects on placental function. These characteristics provide unique insights into fundamental iron-outcome relationships while remaining relevant to low- and middle-income countries experiencing similar dual burdens of iron deficiency and excess [32]. The 2024 US Preventive Services Task Force noted insufficient evidence for optimal iron strategies, and international guidelines are increasingly emphasizing individualized approaches based on ferritin assessment, directly supporting our observation of increased adverse birth outcomes at higher ferritin levels [33,34]. Finally, our baseline provides irreplaceable context for evaluating the long-term impacts of past supplementation policies. The offspring of our cohort, now adults, offer unique opportunities to investigate the developmental origins of health and disease related to maternal iron exposure—insights impossible with recently collected data.

The absence of statistically significant non-linearity in our study contrasts with U-shaped or J-shaped relationships reported in some previous investigations examining maternal iron status and pregnancy outcomes [7,10]. Several factors may explain this difference. First, our population had relatively adequate iron status (median ferritin 26 µg/L, IQR: 16–37), with few women exhibiting severe deficiency (<12 µg/L) that might contribute to adverse outcomes at lower ferritin levels. Widespread prenatal iron supplementation may have prevented severe deficiency that could manifest as the lower limb of a U-shaped curve [7]. Second, we measured ferritin at 30–34 weeks (late third trimester), whereas studies reporting non-linear patterns often assessed iron status in early or mid-pregnancy, when iron requirements and metabolism differ substantially [19,20,35]. Third, modest sample sizes per quintile (*n* = 64–77) may have limited statistical power to detect associations at lower ferritin levels. The monotonic pattern has important implications. Unlike a U-shaped relationship requiring careful balance between deficiency and excess [7], our monotonic pattern within the observed ferritin range indicates higher levels consistently predict greater SGA risk. This supports using continuous modeling approaches (trend tests, per-SD estimates) for population-level inferences and meta-analyses, as these appropriately capture the progressive association [3]. Simultaneously, categorical analysis provides clinical context by identifying markedly elevated ferritin (>43.0 µg/L) as particularly high risk (OR = 3.31, 95% CI: 1.51–7.28), offering a potential threshold for enhanced monitoring. In contrast to some previous research, we found no significant association between ferritin levels and preterm birth. This inconsistency may reflect population-specific differences in anemia prevalence, supplementation protocols, or variations in study gestational age windows. Our study population had relatively low rates of severe anemia, as well as a different gestational age window compared to populations in previous studies where associations with preterm birth were observed [36,37].

The observed reduction in placental weight among women with elevated ferritin offers a plausible mechanistic explanation for the increased SGA risk. Placental weight reflects placental size, vascularization, and nutrient transfer capacity [38]; thus, reduced placental mass may limit fetal nutrient supply and growth. Unlike the gradual, dose-dependent pattern seen for SGA, the placental response appeared to follow a threshold pattern—adverse effects emerged only once ferritin exceeded approximately 43 µg/L. This suggests that beyond a critical ferritin level, excess iron or ferritin-related inflammation may impair placental development and function. The significant positive association we observed between placental weight and both birth weight Z-score and centile supports this concept, suggesting that reduced placental mass may mediate the relationship between elevated ferritin and SGA.

The association between high maternal ferritin levels and SGA births suggests that excess iron or ferritin elevation due to inflammation may impair fetal growth. Multiple biological mechanisms could explain this link. Elevated ferritin without high serum iron signals inflammation, which activates cytokines, impairing placental function and restricting fetal growth [25]. Our findings may support the inflammation hypothesis: high ferritin inversely correlates with placental measurements, implying compromised placental development. Elevated ferritin also indicates oxidative stress, where excess iron produces reactive oxygen species, potentially damaging placental tissue and hindering nutrient transfer, potentially leading to SGA. Then, elevated ferritin may reflect altered maternal-fetal iron homeostasis. Functional iron deficiency, with high ferritin but low transferrin saturation, could reduce iron bioavailability for fetal oxygenation, potentially causing fetal hypoxia and growth impairment [27]. Recent research links excessive placental iron accumulation to impaired trophoblast function and angiogenesis, contributing to adverse pregnancy outcome [39]. The placenta regulates fetal iron via specific pathways; their disruption can lead to suboptimal iron transfer [40]. This complex relationship underscores the importance of balanced iron status and careful maternal iron biomarker monitoring for optimal fetal growth.

This study has several strengths. Comprehensive dose–response analysis using quintile-based categorization, combined with rigorous non-linearity testing, confirms a monotonic ferritin–SGA relationship. Integration of placental parameters allows mechanistic exploration of how elevated ferritin may impair fetal growth through reduced placental mass. The findings highlight the importance of third-trimester ferritin monitoring for identifying pregnancies at risk of adverse outcomes. Several limitations merit consideration. First, convenience sampling yielded a cohort with lower birthweight and higher nulliparity than non-participants, potentially limiting generalizability to low-risk populations. Second, lack of inflammatory markers (CRP, IL-6) prevents distinguishing ferritin elevation from inflammation versus true iron overload [41]. Third, unmeasured confounding from diet, micronutrient status, infections, genetics, and hepcidin is a key regulator of iron metabolism—may exist [42]. Fourth, data from 1989–1990 predate universal supplementation and modern obesity prevalence [31], though recent studies validate similar dose–response patterns [10,11]. While the 35-year temporal distance means supplementation practices, dietary patterns, and maternal characteristics have evolved substantially, the fundamental biological pathways linking iron status to placental function remain conserved. Finally, modest sample sizes per quintile (*n* = 64–77) limited power to detect associations at lower ferritin ranges. Despite these limitations, our integration of placental parameters with rigorous non-linearity testing provides novel mechanistic insights into the ferritin–SGA relationship.

This finding challenges the conventional paradigm that focuses solely on iron deficiency during pregnancy and highlights that iron excess or dysregulated iron metabolism can also be detrimental. By capturing the full ferritin spectrum from deficient to elevated levels our analysis provides a more comprehensive view of the ferritin birth outcome relationship, which is particularly relevant for Asian populations with heterogeneous iron supplementation practices. The monotonic nature of this relationship supports the validity of using both categorical (for clinical interpretation) and continuous (for standardization) analytical approaches. Notably, this is particularly relevant given that the recommendations for universal iron supplementation during pregnancy may not be optimal for all women [43]. Future research should use a broader iron biomarker panel (hepcidin, transferrin saturation, serum iron) to characterize iron metabolism and differentiate iron states [44]. Investigating inflammatory markers and placental histopathology will clarify ferritin’s role in inflammation and its link to fetal growth restriction [45]. Additionally, future studies should examine iron status trajectories from preconception through postpartum, with placental gene expression analysis, to clarify maternal iron dysregulation’s long-term consequences. Validation of our findings in contemporary populations with comprehensive inflammatory marker assessment would strengthen generalizability. Investigating the link between elevated ferritin and SGA is crucial for understanding offspring health, neurodevelopment, and chronic disease risk.

## 5. Conclusions

Our study demonstrates that increasing maternal ferritin levels in the third trimester are associated with a dose-dependent increase in SGA risk, while ferritin levels above 43 µg/L are linked to reduced placental weight. These findings highlight the importance of balancing iron status during pregnancy addressing both deficiency and excess. Routine ferritin monitoring and individualized iron supplementation strategies, rather than universal protocols, may help optimize placental function and fetal growth.

## Figures and Tables

**Table 1:** Maternal and Infants demographic characteristics and laboratory data.

Characteristics	Study Participants	Non-Study Cohort	*p*
	362	1822	
**Maternal Characteristics**			
**Age (years)**			
<20 years	27 (7.5)	156 (8.6)	0.685
20–35 years	324 (89.5)	1620 (88.9)	
>35 years	11 (3.0)	46 (2.5)	
**Pre-pregnancy BMI (kg/m^2^) ^A^**			
Underweight (<18.5)	44 (12.2)	184 (10.1)	0.230
Normal weight (18.5–22.9)	252 (69.6)	1172 (64.3)	
Overweight (23.0–24.9)	37 (10.2)	265 (14.6)	
Obese (≥25.0)	29 (8.0)	201 (11.0)	
**Occupation**			
Agriculture	58 (16.0)	265 (14.5)	0.498
Employee	64 (17.7)	334 (18.3)	
Government official and Enterprise official	52 (14.4)	344 (18.9)	
Trader	29 (8.0)	129 (7.1)	
Handicraft	32 (8.8)	136 (7.5)	
Unemployed women	109 (30.1)	518 (28.4)	
Others	18 (5.0)	96 (5.3)	
**Education**			
No education and Primary school	259 (71.5)	1168 (64.1)	**0.007**
Secondary school or higher	103 (28.5)	654 (35.9)	
**Parity**			
Nulliparous women	236 (65.2)	1026 (56.3)	**0.002**
Parous women	126 (34.8)	796 (43.7)	
**Smoking before Pregnancy**	12 (3.3)	30 (1.7)	0.075
**Alcohol consuming before pregnancy**	3 (0.8)	15 (0.8)	0.992
**Fetal Characteristics**			
Gestational age at delivery (weeks)	39 (38, 40)	39 (38, 40)	0.778
Birthweight (grams)	2850 (2420, 3060)	3050 (2780, 3320)	**<0.001**
**Infant sex**			
Male	842 (46.2)	189 (52.2)	0.583
Female	980 (53.8)	173 (47.8)	
**Mode of delivery**			
Vaginal delivery	1628 (89.3)	324 (89.5)	0.663
Cesarian section	194 (10.7)	38 (10.5)	

Continuous data are reported as median (IQR: quartile 1, quartile 3) and categorical data as *n* (%); ^A^ BMI (body mass index) classification based on the Asia-Pacific criteria; *p*-values were calculated using Mann–Whitney U test for continuous variables and Chi-square test (or Fisher’s exact test where appropriate) for categorical variables; Statistically significant differences (*p* < 0.05) are indicated in bold.

**Table 2:** Maternal Iron Status Across Preterm/Term Births, and Small/Non-Small for Gestational Age Groups by the Third Trimester of Gestation.

		Preterm Birth ^A^	Term Birth	SGA ^B^	Non-SGA
*n* (%)		45 (12.4)	317 (87.6)	120 (33.2)	242 (66.8)
median (IQR)		36 (35, 36)	39 (38, 40)	3.7 (1.4, 6.5)	30.0 (19.8, 46.6)
**Iron status parameters**					
Ferritin (µg/L)					
median (IQR)		31 (17, 41)	23 (14, 38)	26 (17, 48)	23 (12, 37)
range		5–149	3–312	3–312	3–270
Serum Iron (µg/dL)					
median (IQR)		101 (68, 127)	94 (71, 112)	96 (77, 117)	92 (68, 112)
range		29–208	21–284	32–284	21–236
Total iron binding capacity (µmol/L)					
median (IQR)		462 (420, 543)	497 (435, 561)	497 (442, 545)	497 (423, 568)
range		277–822	70–847	279–726	70–847
Transferrin Saturation (%)					
median (IQR)		18.6 (14.2, 31.8)	18.3 (13.8, 23.8)	19.5 (15.0, 24.6)	17.7 (13.3, 24.3)
range		5.1–66.7	5.0–100	7.0–62.7	5.0–100
Mean Cell Hemoglobin Concentration					
median (IQR)		32.0 (30.9, 32.8)	32.1 (31.1, 32.9)	32.0 (31.0, 32.8)	32.2 (31.2, 33.0)
range		27.3–34.4	28.8–37.1	27.3–35.6	28.8–37.0
Hemoglobin (g/dL)					
median (IQR)		11.6 (11.0, 12.41)	11.5 (11.0, 12.1)	11.4 (11.0, 12.3)	11.6 (11.0, 12.1)
range		7.8–13.5	9.2–13.9	9.1–13.7	8.6–13.8

Values are presented as median (interquartile range) with minimum-maximum ranges provided for each parameter. Blood samples were collected at approximately 32 weeks (third trimester, range 30–34 weeks) of gestation. ^A^ Preterm birth was defined as delivery before 37 completed weeks of gestation. ^B^ Small for gestational age (SGA) was defined as birth weight below the 10th percentile for gestational age using population-specific growth charts.

**Table 3:** Odds Ratios (95% Confidence Intervals) for Preterm Birth and Small for Gestational Age.

Outcome	Serum Ferritin at 30–34th Weeks of Gestation (µg/L)					*p* Trend	Per-SD Increase
	Quintiles 1 (<13.0)	Quintiles 2 (13.0–20.0)	Quintiles 3 (20.1–30.0)	Quintiles 4 (30.1–43.0)	Quintiles 5 (>43.0)		
*n* (%)	77 (21.3)	72 (19.9)	77 (21.3)	64 (17.7)	72 (19.9)		
**Preterm birth**							
Rate: *n* (%)	6 (7.8)	10 (13.9)	6 (7.8)	12 (18.8)	11 (15.3)		
Crude OR (95% CI)	0.45 (0.17, 1.21)	0.72 (0.29, 1.79)	0.37 (0.13, 1.04)	1.00 (Reference)	0.80 (0.33, 1.96)	0.182	1.05 (0.78, 1.40)
Adjusted OR (95% CI)	0.71 (0.24, 2.10)	1.00 (0.37, 2.72)	0.46 (0.15, 1.39)	1.00 (Reference)	1.10 (0.41, 2.96)	0.484	1.01 (0.71, 1.44)
**Small for gestational age**							
Rate: *n* (%)	16 (20.8)	23 (31.9)	30 (39.0)	17 (26.6)	34 (47.2)	**0.001**	
Crude OR (95% CI)	0.88 (0.40, 1.90)	1.63 (0.78, 3.40)	1.98 (0.97, 4.04)	1.00 (Reference)	**3.16** **(1.54, 6.50)**	**0.005**	**1.32** **(1.06, 1.64)**
Adjusted OR (95% CI)	1.06 (0.46, 2.44)	1.72 (0.78, 3.82)	2.04 (0.94, 4.41)	1.00 (Reference)	**3.31** **(1.51, 7.28)**	**0.044**	**1.31** **(1.01, 1.71)**

Logistic regression models were adjusted for maternal age, pre-pregnancy maternal BMI, maternal education, and parity; OR odds ratio; CI, confidence interval; Bold values indicate statistical significance (*p* < 0.05).

**Table 4:** Association Between Elevated Maternal Serum Ferritin Levels (>43.0 µg/L) and Placental Characteristics in the Third Trimester of Pregnancy.

Placental Parameter	Category	Range	*n* (%)	Crude OR (95% CI)	*p*	Adjusted OR (95% CI)	*p*
**Placental Weight (grams)**	Low (<−1 SD)	<415	61 (16.9)	3.27 (1.77, 6.05)	**<0.001**	3.02 (1.61, 5.69)	**0.001**
	Normal (−1 SD to +1 SD)	415–629	248 (68.5)	1.00 (Reference)	-	1.00 (Reference)	-
	High (>+1 SD)	>629	53 (14.6)	0.77 (0.32, 1.82)	0.549	0.80 (0.33, 1.93)	0.615
**Placental** **Diameter** **(cm)**	Small (<−1 SD)	<14.5	13 (3.6)	1.16 (0.31, 4.35)	0.821	1.02 (0.27, 3.89)	0.982
	Normal (−1 SD to +1 SD)	14.5–23.8	332 (91.7)	1.00 (Reference)	-	1.00 (Reference)	-
	Large (>+1 SD)	>23.8	17 (4.7)	0.24 (0.03, 1.86)	0.173	0.25 (0.03, 1.97)	0.190
**Umbilical Cord Length (cm)**	Short (<−1 SD)	<38.9	47 (13.0)	1.39 (0.67, 2.87)	0.371	1.31 (0.63, 2.74)	0.467
	Normal (−1 SD to +1 SD)	38.9–58.3	253 (69.9)	1.00 (Reference)	-	1.00 (Reference)	-
	Long (>+1 SD)	>58.3	62 (17.1)	0.78 (0.37, 1.64)	0.515	0.90 (0.42, 1.93)	0.791

Multinomial logistic regression models were adjusted for maternal age, pre-pregnancy maternal BMI, maternal education, and parity; OR, odds ratio; CI, confidence interval; Bold values indicate statistical significance (*p* < 0.05); The reference category for each placental parameter represents measurements within normal range (−1 SD to +1 SD).

**Table 5:** Association Between Placental Characteristics and Standardized Infant Birth Weight Outcomes.

Placental Parameter	Birth Weight Z-Score		Birth Weight Centile	
	*β* Coefficient (95% CI)	*p*	*β* Coefficient (95% CI)	*p*
Placental Weight (per 100 g)	0.39 (0.32, 0.47)	**<0.001**	8.58 (6.78, 10.37)	**<0.001**
Placental Diameter (per cm)	0.02 (−0.01, 0.03)	0.075	0.24 (−0.14, 0.62)	0.219
Umbilical Cord Length (per cm)	0.00 (−0.01, 0.01)	0.107	−0.15 (−0.35, 0.04)	0.121

Multiple linear regression models were adjusted for maternal age, pre-pregnancy maternal BMI, maternal education, and parity; *β*, coefficients; CI, confidence interval; Bold values indicate statistical significance (*p* < 0.05).

## Data Availability

The data supporting this study is available within the article. Raw data supporting this study’s findings are available from the corresponding author upon reasonable request.

## Supplementary Materials

The following supporting information can be downloaded at: https://www.mdpi.com/article/10.3390/nu17243911/s1, Table S1: Tests for Non-linearity in the Ferritin and Small for Gestational Age Relationship.

## References

[1] Hamner H.C., Nelson J.M., Sharma A.J., Jefferds M.E.D., Dooyema C., Flores-Ayala R., Bremer A.A., Vargas A.J., Casavale K.O., de Jesus J.M. (2022). Improving Nutrition in the First 1000 Days in the United States: A Federal Perspective. Am. J. Public Health.

[2] Wang X. (2023). Prenatal Nutrition and Developmental Origins of Health and Disease. Precis. Nutr..

[3] Quezada-Pinedo H.G., Cassel F., Duijts L., Muckenthaler M.U., Gassmann M., Jaddoe V.W.V., Reiss I.K.M., Vermeulen M.J. (2021). Maternal Iron Status in Pregnancy and Child Health Outcomes After Birth: A Systematic Review and Meta-Analysis. Nutrients.

[4] Ataide R., Fielding K., Pasricha S.R., Bennett C. (2023). Iron deficiency, pregnancy, and neonatal development. Int. J. Gynaecol. Obstet..

[5] Sangkhae V., Fisher A.L., Ganz T., Nemeth E. (2023). Iron Homeostasis During Pregnancy: Maternal, Placental, and Fetal Regulatory Mechanisms. Annu. Rev. Nutr..

[6] Daru J., Colman K., Stanworth S.J., De La Salle B., Wood E.M., Pasricha S.R. (2017). Serum ferritin as an indicator of iron status: What do we need to know?. Am. J. Clin. Nutr..

[7] Dewey K.G., Oaks B.M. (2017). U-shaped curve for risk associated with maternal hemoglobin, iron status, or iron supplementation. Am. J. Clin. Nutr..

[8] Fite M.B., Tura A.K., Yadeta T.A., Oljira L., Roba K.T. (2022). Prevalence, predictors of low birth weight and its association with maternal iron status using serum ferritin concentration in rural Eastern Ethiopia: A prospective cohort study. BMC Nutr..

[9] Dande A., Pajai S., Gupta A., Dande S., Sethi N. (2024). Unraveling the Role of Maternal Serum Ferritin Levels in Preterm Delivery: A Comprehensive Review. Cureus.

[10] Tao Y., Kang J., Liu J., Duan J., Wang F., Shi Y., Li Y., Wang C., Xu D., Qu X. (2022). Association of low birthweight and small for gestational age with maternal ferritin levels: A retrospective cohort study in China. Front. Nutr..

[11] Yang L., Wu L., Liu Y., Chen H., Wei Y., Sun R., Shen S., Zhan B., Yang J., Deng G. (2022). Association Between Serum Ferritin Concentration and Risk of Adverse Maternal and Fetal Pregnancy Outcomes: A Retrospective Cohort Study. Diabetes Metab. Syndr. Obes..

[12] Chawla D. (2019). Small for Gestation Age Neonates: Unmet Clinical Care and Research Need. Indian J. Pediatr..

[13] Lawn J.E., Ohuma E.O., Bradley E., Idueta L.S., Hazel E., Okwaraji Y.B., Erchick D.J., Yargawa J., Katz J., Lee A.C.C. (2023). Small babies, big risks: Global estimates of prevalence and mortality for vulnerable newborns to accelerate change and improve counting. Lancet.

[14] Hwang I.T. (2019). Long-term care, from neonatal period to adulthood, of children born small for gestational age. Clin. Pediatr. Endocrinol..

[15] Gnawali A. (2021). Prematurity and the Risk of Development of Childhood Obesity: Piecing Together the Pathophysiological Puzzle. A Literature Review. Cureus.

[16] Applegate J.A., Islam M.S., Khanam R., Roy A.D., Chowdhury N.H., Ahmed S., Mitra D.K., Mahmud A., Islam M.S., Saha S.K. (2024). Young Infant Mortality Associated with Preterm and Small-for-Gestational-Age Births in Rural Bangladesh: A Prospective Cohort Study. J. Pediatr..

[17] Roberts H., Woodman A.G., Baines K.J., Jeyarajah M.J., Bourque S.L., Renaud S.J. (2021). Maternal Iron Deficiency Alters Trophoblast Differentiation and Placental Development in Rat Pregnancy. Endocrinology.

[18] Zhang Y., Lu Y., Jin L. (2022). Iron Metabolism and Ferroptosis in Physiological and Pathological Pregnancy. Int. J. Mol. Sci..

[19] Broekhuis A., Koenen S.V., Broeren M.A.C., Krabbe J.G., Pop V.J.M. (2024). High first trimester ferritin levels differ according to parity and are independently related to preterm birth: A prospective cohort study. Acta Obstet. Gynecol. Scand..

[20] Zhou H., Lu Y., Luo J., Pan B., Zhao Q., Chen M., Ma Z.F. (2025). Maternal iron deficiency assessed by serum ferritin and birth outcomes in mainland China. Sci. Rep..

[21] Mangklabruks A., Rerkasem A., Wongthanee A., Rerkasem K., Chiowanich P., Sritara P., Pruenglampoo S., Yipintsoi T., Tongsong T., Marshall T. (2012). The risk factors of low birth weight infants in the northern part of Thailand. J. Med. Assoc. Thail..

[22] Papageorghiou A.T., Kennedy S.H., Salomon L.J., Altman D.G., Ohuma E.O., Stones W., Gravett M.G., Barros F.C., Victora C., Purwar M. (2018). The INTERGROWTH-21(st) fetal growth standards: Toward the global integration of pregnancy and pediatric care. Am. J. Obstet. Gynecol..

[23] International Committee for Standardization in Haematology (ICSH) (1981). The theory of reference values. Clin. Lab. Haematol..

[24] Behrouzi-Lak T., Mortazavi M., Vazifeshenas S. (2021). Maternal Serum Ferritin Level in Prediction of Mothers with Appropriate-For-Gestational-Age (AGA), Small-For-Gestational Age (SGA), and Intrauterine Growth Restriction (IUGR). J. Pediatr. Perspect..

[25] Mégier C., Peoc’h K., Puy V., Cordier A.G. (2022). Iron Metabolism in Normal and Pathological Pregnancies and Fetal Consequences. Metabolites.

[26] Yu Y., Woloshun R.R., Lee J.K., Ebea P.O., Zhu S., Nemeth E., Garrick L.M., Garrick M.D., Collins J.F. (2023). Fetal factors disrupt placental and maternal iron homeostasis in murine β-thalassemia. Blood.

[27] Cacoub P., Vandewalle C., Peoc’h K. (2019). Using transferrin saturation as a diagnostic criterion for iron deficiency: A systematic review. Crit. Rev. Clin. Lab. Sci..

[28] Yang J., Chang Q., Du Q., Liu X., Dang S., Tian X. (2024). Maternal iron nutrition during pregnancy and fetal intrauterine growth. Nutr. J..

[29] Banerjee A., Athalye S., Shingade P., Khargekar V., Mahajan N., Madkaikar M., Khargekar N. (2024). Efficacy of daily versus intermittent oral iron supplementation for prevention of anaemia among pregnant women: A systematic review and meta-analysis. eClinicalMedicine.

[30] Kent L., McGirr M., Eastwood K.A. (2024). Global trends in prevalence of maternal overweight and obesity: A systematic review and meta-analysis of routinely collected data retrospective cohorts. Int. J. Popul. Data Sci..

[31] Ratnasiri A.W.G., Lee H.C., Lakshminrusimha S., Parry S.S., Arief V.N., DeLacy I.H., Yang J.S., DiLibero R.J., Logan J., Basford K.E. (2019). Trends in maternal prepregnancy body mass index (BMI) and its association with birth and maternal outcomes in California, 2007–2016: A retrospective cohort study. PLoS ONE.

[32] Winichagoon P., Margetts B.M., Romieu I., Dossus L., Willett W.C. (2017). The double burden of malnutrition in low- and middle-income countries. Energy Balance and Obesity.

[33] Nicholson W.K., Silverstein M., Wong J.B., Chelmow D., Coker T.R., Davis E.M., Jaén C.R., Krousel-Wood M., Lee S., Li L. (2024). Screening and Supplementation for Iron Deficiency and Iron Deficiency Anemia During Pregnancy: US Preventive Services Task Force Recommendation Statement. JAMA.

[34] O’Toole F., Sheane R., Reynaud N., McAuliffe F.M., Walsh J.M. (2024). Screening and treatment of iron deficiency anemia in pregnancy: A review and appraisal of current international guidelines. Int. J. Gynaecol. Obstet..

[35] Roemhild K., von Maltzahn F., Weiskirchen R., Knüchel R., von Stillfried S., Lammers T. (2021). Iron metabolism: Pathophysiology and pharmacology. Trends Pharmacol. Sci..

[36] Beressa G., Whiting S.J., Kuma M.N., Lencha B., Belachew T. (2024). Association between anemia in pregnancy with low birth weight and preterm birth in Ethiopia: A systematic review and meta-analysis. PLoS ONE.

[37] Khezri R., Salarilak S., Jahanian S. (2023). The association between maternal anemia during pregnancy and preterm birth. Clin. Nutr. ESPEN.

[38] Munaf Z., Baloch F.N., Bosan R., Ismail A., Punar Z.A., Khan S. (2025). Correlation Between Placenta Weight and Birth Weight at Full Term Pregnancy: Placenta Weight and Birth Weight Correlation. Pak. J. Health Sci..

[39] Ng S.W., Norwitz S.G., Norwitz E.R. (2019). The Impact of Iron Overload and Ferroptosis on Reproductive Disorders in Humans: Implications for Preeclampsia. Int. J. Mol. Sci..

[40] Lakhal-Littleton S. (2021). Advances in understanding the crosstalk between mother and fetus on iron utilization. Semin. Hematol..

[41] Liao Y., Zeng T., Guo X., Li X. (2025). Ferritin’s role in infectious diseases: Exploring pathogenic mechanisms and clinical implications. New Microbes New Infect..

[42] Bardan C.R., Ionita I., Iordache M., Lighezan D., Gluhovschi A., Enatescu I., Bernad E. (2024). Hepcidin as the Central Regulator in Pregnancy-Associated Iron Deficiency Anemia and Vitamin D Deficiency. Timis. Med. J..

[43] Finkelstein J.L., Cuthbert A., Weeks J., Venkatramanan S., Larvie D.Y., De-Regil L.M., Garcia-Casal M.N. (2024). Daily oral iron supplementation during pregnancy. Cochrane Database Syst. Rev..

[44] Pavord S., Daru J., Prasannan N., Robinson S., Stanworth S., Girling J. (2020). UK guidelines on the management of iron deficiency in pregnancy. Br. J. Haematol..

[45] Vornic I., Buciu V., Furau C.G., Gaje P.N., Ceausu R.A., Dumitru C.S., Barb A.C., Novacescu D., Cumpanas A.A., Latcu S.C. (2024). Oxidative Stress and Placental Pathogenesis: A Contemporary Overview of Potential Biomarkers and Emerging Therapeutics. Int. J. Mol. Sci..

