# Supplementary material for: Maternal Serum Ferritin Levels in Third Trimester and Risk of Small for Gestational Age in Northern Thailand: Implications for Management in Pregnancy
Source: Nutrients. 2025 Dec 13;17(24):3911. doi: 10.3390/nu17243911 (PMC12735463; doi:10.3390/nu17243911)
Supplement: Supplementary file 1 [file nutrients-17-03911-s001.zip › nutrients-3958910-supplementary.pdf]

Supplementary data

Supplementary Materials Table S1. Tests for Non-linearity in the Ferritin and Small for Gestational Age Relationship.

Test	Chi-sqaure	*p*	Interpretation
**Wald test (quadratic term)** [A]	$\chi^2 = 0.62$	0.432	Tests if quadratic term ≠ 0
**Likelihood ratio test** [B]	LR $\chi^2 = 0.59$	0.443	Compares linear vs. quadratic models
**Restricted cubic spline** [C] **(df=3)**	$\chi^2 = 1.10$	0.575	Tests non-linear spline terms

All models were adjusted for maternal age, pre-pregnancy maternal BMI, parity, Serum iron, Total iron binding capacity, Transferrin saturation, Corpuscular Hemoglobin Concentration, Hemoglobin; OR odds ratio; CI, confidence interval; Bold values indicate statistical significance ($p < 0.05$).

[A] Wald test: Tests statistical significance of the quadratic term

[B] Likelihood ratio test: Compares model fit between linear and quadratic models

[C] Restricted cubic spline: Uses 4 knots placed at 5th, 35th, 65th, and 95th percentiles of ferritin distribution
